# COVID-19 Triage and Test Center: Safety, Feasibility, and Outcomes of Low-Threshold Testing

**DOI:** 10.3390/jcm9103217

**Published:** 2020-10-07

**Authors:** Gregory Mansella, Marco Rueegg, Andreas F. Widmer, Sarah Tschudin-Sutter, Manuel Battegay, Julia Hoff, Kirstine K. Søgaard, Adrian Egli, Bram Stieltjes, Karoline Leuzinger, Hans H. Hirsch, Andrea Meienberg, Thilo Burkard, Michael Mayr, Roland Bingisser, Christian H. Nickel

**Affiliations:** 1 Emergency Department, University Hospital Basel, University of Basel, CH-4031 Basel, Switzerland; gregory.mansella@usb.ch (G.M.); marco.rueegg@usb.ch (M.R.); juliamaria.hoff@usb.ch (J.H.); christian.nickel@usb.ch (C.H.N.); 2 Division of Infectious Diseases and Hospital Epidemiology, University Hospital Basel, University of Basel, CH-4031 Basel, Switzerland; andreas.widmer@usb.ch (A.F.W.); sarah.tschudin@usb.ch (S.T.-S.); manuel.battegay@usb.ch (M.B.); hans.hirsch@usb.ch (H.H.H.); 3 Division of Clinical Bacteriology and Mycology, University Hospital Basel, University of Basel, CH-4031 Basel, Switzerland; kirstinekobberoee.soegaard@usb.ch (K.K.S.); adrian.egli@usb.ch (A.E.); 4 Applied Microbiology Research, Department Biomedicine, University of Basel, CH-4031 Basel, Switzerland; 5 Department of Radiology, University Hospital Basel, University of Basel, CH-4031 Basel, Switzerland; bram.stieltjes@usb.ch; 6 Division of Clinical Virology, University Hospital Basel, CH-4031 Basel, Switzerland; karoline.leuzinger@usb.ch; 7 Transplantation and Clinical Virology, Department Biomedicine, University of Basel, CH-4031 Basel, Switzerland; 8 Medical Outpatient Department, University Hospital Basel, University of Basel, CH-4031 Basel, Switzerland; andrea.meienberg@usb.ch (A.M.); thilo.burkard@usb.ch (T.B.); michael.mayr@usb.ch (M.M.)

**Keywords:** COVID-19, symptoms, SARS-CoV-2, Triage and Test Center, disposition, resource allocation, drive-thru testing

## Abstract

This prospective observational study evaluated the safety and feasibility of a low threshold testing process in a Triage and Test Center (TTC) during the early course of the coronavirus disease 19 (COVID-19) pandemic. In addition, we aimed to identify clinical predictors for a positive severe acute respiratory syndrome coronavirus 2 (SARS-CoV-2) swab result. Patients underwent informal triage, standardized history taking, and physician evaluation, only where indicated. Patients were observed for 30 days. Safety was the primary outcome and was defined as a COVID-19-related 30 day re-presentation rate <5% and mortality rate <1% in patients presenting to the TTC. Feasibility was defined as an overruling of informal triage <5%. Among 4815 presentations, 572 (11.9%) were tested positive for SARS-CoV-2, and 4774 were discharged. Mortality at 30-days was 0.04% (2 patients, one of which related to COVID-19). Fever (OR 2.03 [95% CI 1.70;2.42]), myalgia (OR 1.94 [1.63;2.31]), chills (OR 1.77 [1.44;2.16]), headache (OR 1.61 [1.34;1.94]), cough (OR 1.50 [1.24;1.83]), weakness (OR 1.46 [1.21;1.76]), and confusion (OR 1.39 [1.06;1.80]) were associated with test positivity. Re-presentation rate was 8% overall and 1.4% in COVID-19 related re-presentation (69 of 4774). The overruling rate of informal triage was 1.5%. According to our study, a low-threshold testing process in a TTC appeared to be safe (low re-presentation and low mortality) and is feasible (low overruling of informal triage). A COVID-19 diagnosis based on clinical parameters only does not appear possible.

## 1. Introduction

Regular, widespread testing to identify and isolate individuals with coronavirus disease 19 (COVID-19) appears to be crucial to reduce transmission [1,2], but limitations on testing availability has been a challenge during the early phase of COVID-19 pandemic. To implement a low-threshold testing, we setup a Triage and Test Center (TTC) in the early phase of the pandemic. Expecting a high number of patients willing to be tested, we designed a quick and easy walk-in process. This process was based on previous safety data regarding informal triage and physiological reserve (e.g., mobility). In detail, unimpaired mobility, low physician disease severity ratings (PDSR), and normal vital signs are associated with a very favorable prognosis [3,4,5,6,7]. We hypothesized that these principles hold true for a coordinated large-scale testing process. Several similar approaches, such as a drive-thru process in COVID-19 testing have been described, but to date, safety data are lacking [8].

In the present study, we evaluated feasibility and safety of a Triage and Test Center for low-threshold testing, to determine patient outcomes, and to identify clinical predictors for a positive nasopharyngeal SARS-CoV-2 swab result.

## 2. Methods

The emergency department (ED) of the University Hospital Basel (UHBS), a tertiary care center, has a yearly census of 54,000 patients. UHBS serves as a major provider for a population of about 176,000 inhabitants and is the designated COVID-19 hospital for the Canton (State). The TTC was located in a church in close vicinity (entrance 30 m from the ED front door). A signpost diverted patients with flu-like symptoms from the ED and other front doors to the TTC. To ensure social distancing while waiting for testing, a waiting area supervised by security staff was marked outside of the TTC. Opening hours were from 8 am to 11 pm. The TTC was designed to provide a maximal test capacity of 500 tests daily. A simulation was performed prior to the launch of the TTC to ensure patient-flow and safety. We briefed team members before each shift in adherence to personal protective equipment guidelines, the use of informal triage, and test administration. Health authorities endorsed testing in symptomatic patients and advertised the location through media (newspaper, internet, radio, television, and social media). After evaluation in the TTC, patients deemed in need of further work-up were transferred to the ED. We established a notification pathway for providing test results through text messaging to patients. Local health authorities were informed automatically about all tested cases. Patients were instructed to present to the ED if their symptoms worsened. We performed patient follow-ups for COVID-19 positive cases.

This study was approved by the local ethics committee (EKNZ-identifier: 2020-00790 and 2020-00769). The study is reported according to Strobe guidelines.

### 2.1. Study Population

The eligible population were consecutive patients who presented and met testing criteria for COVID-19, including those with respiratory symptoms (such as shortness of breath), other flu-like symptoms (such as fever, sore throat, or cough) of any degree, and/or self-reported exposure to COVID-19.

### 2.2. Methods of Measurement

At the TTC front door, a senior physician screened patients for symptoms and acuity. Informal triage using physician disease severity rating (PDSR) was used to identify patients in need of a clinical evaluation. PDSR was based on a very brief subjective first impression, using a numeric rating scale from 0 to 10 points by asking “how ill does this patient look” (0 = not appearing ill at all, 10 = appearing very seriously ill). PDSR was previously shown to be non-inferior to formal triage regarding safety outcomes, such as mortality [3]. In case of a PDSR higher than 2/10, complete vital sign assessment was to be performed and the patient was evaluated by a physician. Patients filled out a questionnaire regarding symptoms and comorbidities on a machine-readable case-report form. Previously trained medical students reviewed the questionnaire, and performed swabs [9].

In order to implement a low-threshold testing strategy in the virology lab, we provided cross-validated laboratory-developed and commercial SARS-CoV-2 reverse transcription polymerase chain reaction (RT-PCR) assays detecting the viral S-gene, E-gene, and ORF8-gene sequences including the Roche Cobas 6800 assay (Roche Diagnostics, Rotkreuz, Switzerland) [10,11]. Two swabs from the naso- and oropharyngeal sites (NOPS), respectively, were taken and combined into one universal transport medium (UTM) tube (Copan, Brescia, Italy). Total nucleic acids (TNAs) were extracted from the UTM using the MagNA Pure 96 system (Roche Diagnostics, Rotkreuz, Switzerland). We tested for SARS-CoV-2 RNA using RT-PCR assays targeting specific viral sequences (S-gene, Basel-SCoV2-S-112bp, in-house methods, not commercial kits) and simultaneously ran a commercial assay (E-gene; Roche Diagnostics, Rotkreuz, Switzerland) head-to-head. Concordant positive or negative results were signed out directly, while discordant results were retested using the ORF8-gene assay (Basel-SCoV2-ORF8-97bp) and signed out accordingly. Validation of the Roche Cobas 6800 demonstrated concordant results with the Basel-SCoV2-S-112bp and E-gene assays of 97.0% of 1344 tests, again using BaselSCoV2-ORF8-97bp testing for discordant results [11].

### 2.3. Outcomes

The primary outcome was the safety of the TTC process (performing informal triage in all patients and physician evaluation including vital sign assessment only in patients deemed to be ill, i.e., PDSR >2) [3]. Safety was defined as a re-presentation rate <5% after discharge from the TTC and a mortality rate <1% during the follow-up of 30 days. Re-presentation was defined as any non-elective re-presentation to UHBS with the exception of re-presentation to the TTC for repeated testing. Re-presentations were stratified into related and unrelated re-presentations. Related re-presentation was defined as a COVID-19 case with re-presentation due to symptoms of COVID-19 (like progressive dyspnea, increasing cough or persistent fever) and a patient with newly diagnosed COVID-19 after initial negative testing in the TTC.

Secondary outcomes were feasibility, the description of population and outcomes, and the identification of clinical predictors for a positive nasopharyngeal SARS-CoV-2 swab result. Feasibility was defined as the successful implementation of the process based on informal triage and the inclusion of a student-staffed swab-team [9], an overruling of discharge in patients with PDSR ≤2 below 5%, and the ability to provide the throughput as planned.

Other outcomes were the characterization of the population and the rate of positive swabs for SARS-CoV-2 over time. We chose potential clinical predictors a priori based on available evidence at that time (8th March 2020) [12,13,14,15].

### 2.4. Statistical Analysis

Descriptive statistics were presented as counts and frequencies for categorical data and medians (Min, Max) for metric or ordinal variables. *p*-values corresponded to multivariable logistic regressions. A *p*-value < 0.05 was considered significant. Odds ratios (OR) were calculated with 95% confidence intervals. For metric and ordinal predictors, ORs were presented as the ratios of the odds increasing the predictor by one unit. In forest plots, ORs were expressed increasing the predictor by one IQR (interquartile range). Adjusted ORs were calculated using multivariable logistic regression including age and gender as covariates. We performed all evaluations with the R software language and environment for statistical computing, R foundation for statistical computing, Vienna, Austria, 2020 (R version 4.0.0).

## 3. Results

### 3.1. Characteristics of Study Subjects

**Figure 1:**
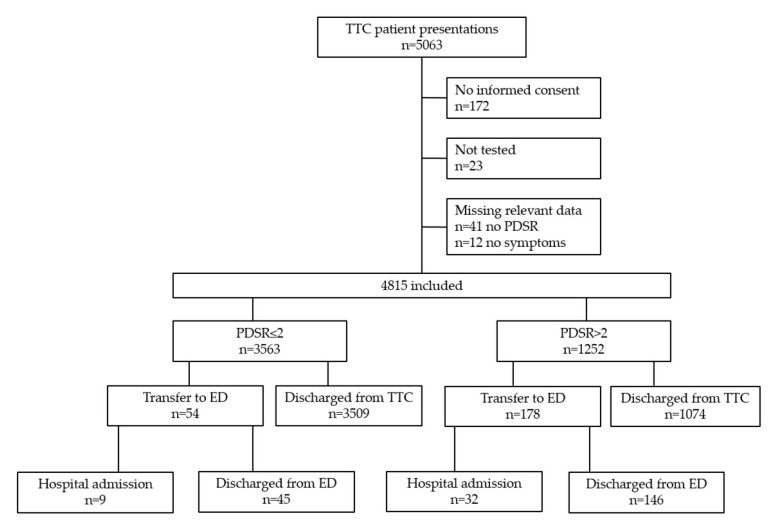
Flowchart of inclusion. TTC = Triage and Test Center; PDSR = Physician Disease Severity Rating; ED = Emergency Department.

Between 19th March and 29th April 2020, 5063 presentations occurred to the TTC. We excluded 248 presentations (4.9%) for the following reasons: 172 patients did not provide informed consent, 23 did not receive a nasopharyngeal swab, and 53 had missing data. Therefore, 4815 presentations were included. 3563 patient presentations (74%) had PDSR ≤2, and 1252 (26%) PDSR >2 (Figure 1).

### 3.2. Safety Outcomes

**Figure 2:**
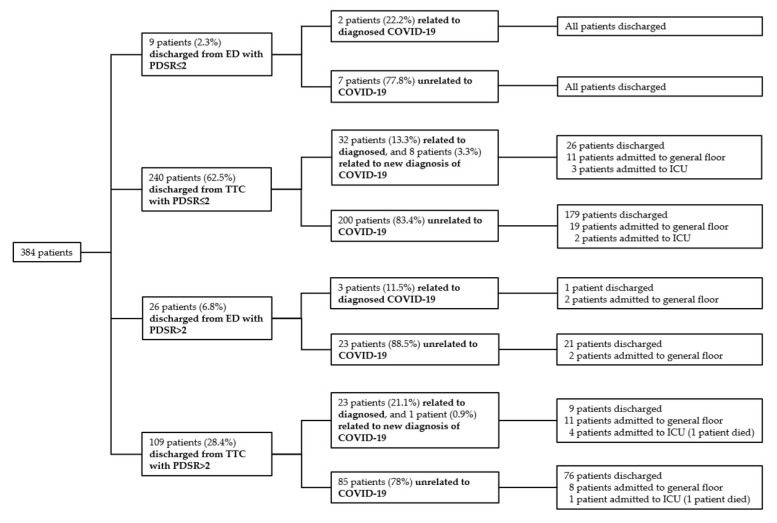
Re-presentations of discharged patients during 30-day follow-up; ED = Emergency Department; TTC = Triage and Test Center; PDSR = Physician Disease Severity Rating; ICU = Intensive Care Unit.

Among all 4774 patient presentations who were discharged (4583 from the TTC (3509 + 1074), 191 from the ED (45 + 146), Figure 1), 384 (8%) patients represented to the hospital during 30-day follow up (Figure 2). Of these, 174 patients (3.6%) represented the ED, 92 patients (1.9%) represented the TTC, and 118 patients (2.5%) represented elective consultation or hospitalization. Most re-presentations were unrelated to COVID-19 (315/384 patients, 82%). Moreover, 69 patients out of 4774 (1.4%) discharged presentations were related to COVID-19. Among these, 9 patients had newly diagnosed COVID-19 (7/92 patients were diagnosed in the TTC, 2/174 patients in the ED). Median time for COVID-19 related and COVID-19 unrelated re-presentations was 6 (min 1, max 29) and 12 (min 1, max 30) days, respectively. Among all included patient presentations, 2 (0.04%) died during 30-day follow-up. Both patients were discharged from the TTC with PDSR 3 (1 patient tested positive and 1 patient tested negative). The first patient, a 58 year-old male, who was tested positive, represented the ED on day 5. He died in the intensive care unit (ICU) of multi-organ failure. The second patient, a 77-year-old female, who tested negative, sustained a ground level fall 29 days after presentation to the TTC. She died postoperatively after a complicated hip fracture. Repeated testing for COVID-19 was negative. Moreover, 13 patients who tested positive for COVID-19 were lost to follow-up. Nine patients lived in another country and 4 patients provided incorrect personal data.

### 3.3. Feasibility of the Triage and Test Center (TTC)

In 54 out of 3563 (1.5%) patient presentations with PDSR ≤2, the standard process of discharge without vital signs and physician examination was overruled by referring patients to the ED (for details, see Table A1). Among these, 9 patients were hospitalized (2 related to COVID-19) and 45 discharged from the ED. Next, 178/1252 (14.2%) presentations with PDSR >2 were transferred to the ED for further evaluation, of which 32 were hospitalized (12 related to COVID-19) and 146 discharged. The majority of all presentations (4583, 95.2%) were discharged from the TTC after a nasopharyngeal swab was performed (Figure 1). During opening hours, 93 patients (min 25, max 358) per day were tested. Most tests were performed on March 19th (first day of the study).

### 3.4. Clinical Predictors for Positive Nasopharyngeal SARS-CoV-2 Swab Result

**Figure A1:**
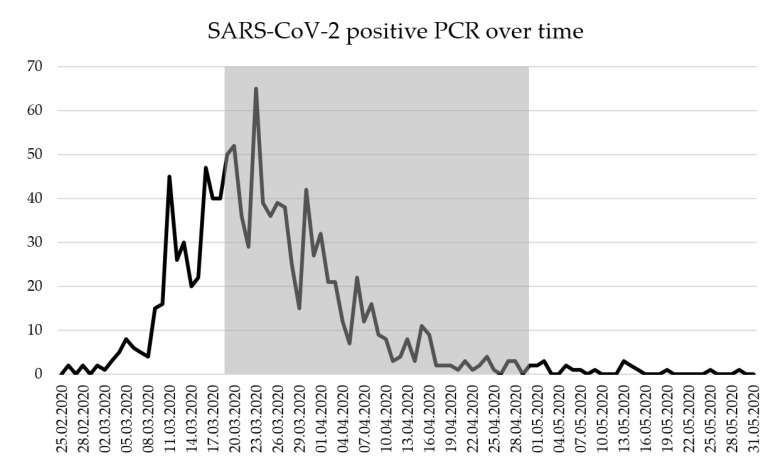
SARS-CoV-2 positive PCR during study period from 19th March to 29th April 2020 (gray marking); SARS-CoV-2 = severe acute respiratory syndrome coronavirus 2; PCR = Polymerase Chain Reaction.

**Figure A2:**
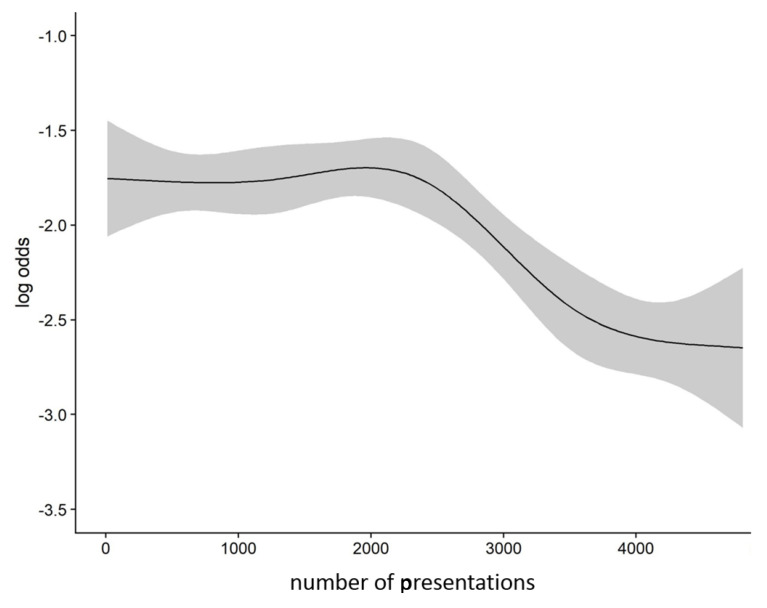
Odds for positive SARS-CoV-2-testing during study period; SARS-CoV-2 = severe acute respiratory syndrome coronavirus 2.

Among all presentations, 572 (11.9%) had a positive swab for SARS-CoV-2, 4243 (88.1%) were negative (Table 1). Most positive testing occurred in the first 3 weeks of the study and started to decline after inclusion of about 2500 presentations (Figure A1 and Figure A2).

**Figure 3:**
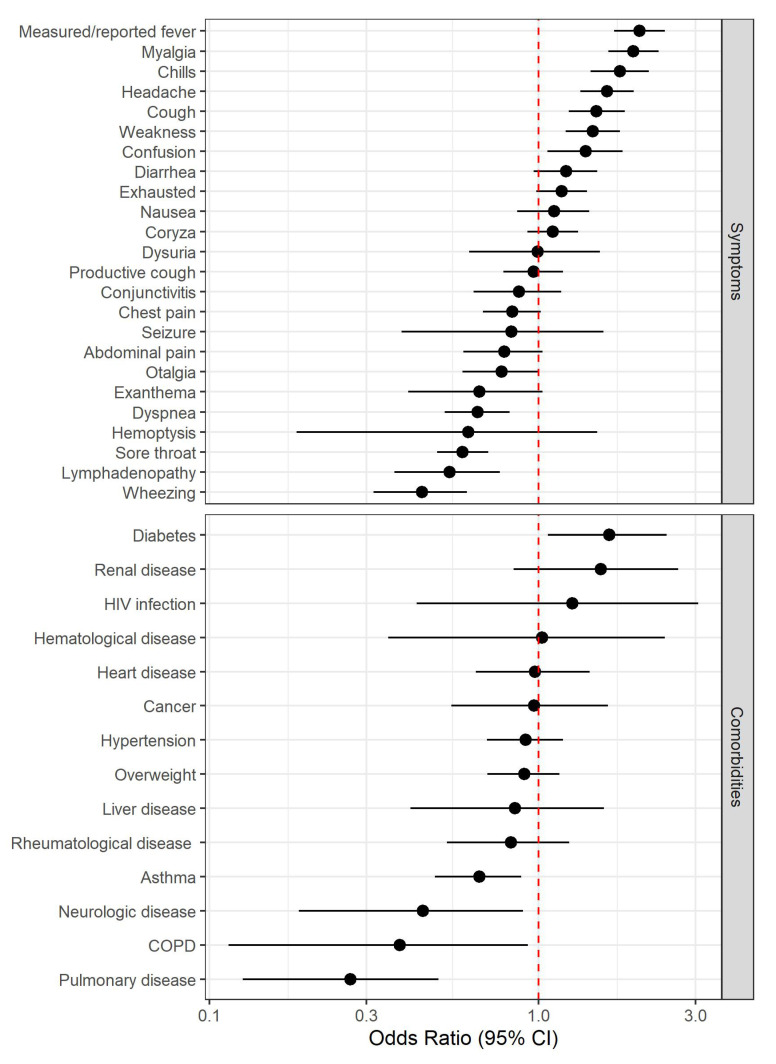
Forest plot for symptoms and comorbidities. COPD = Chronic Obstructive Pulmonary Disease.

The most common reported symptom was cough (3132 presentations, 65%), followed by sore throat (2694, 56%), headache (2693, 55.9%), and coryza (2575, 53.5%). In positive cases, the most common symptom was cough (415, 72.6%), followed by headache (366, 64%), measured/reported fever (323, 56.5%), and coryza (314, 54.9%). Among the 572 positive cases, 192 (33.6%) reported symptoms for seven or more days. Patients with positive swab results were older (OR 1.31 [95% CI 1.15;1.50]). The symptoms most strongly associated with positive tests were measured/reported fever (OR 2.03 [95% CI 1.70;2.42]), myalgia (OR 1.94 [95% CI 1.63;2.31]), chills (OR 1.77 [95% CI 1.44;2.16]), headache (OR 1.61 [1.34;1.94]), and cough (OR 1.50 [95% CI 1.24;1.83]). Wheezing (OR 0.44 [95% CI 0.32;0.61]), lymphadenopathy (OR 0.54 [95% CI 0.37;0.76]), sore throat (OR 0.59 [95% CI 0.49;0.70]), and dyspnea (OR 0.65 [95% CI 0.52;0.82]) were inversely associated with positive tests. Regarding comorbidities, diabetes (OR 1.64 [95% CI 1.07;2.450]) was associated with a positive test, whereas pulmonary disorders (OR 0.27 [95% CI 0.13;0.50]) were inversely associated with a positive test (Figure 3).

Regarding vital signs, elevated temperature (OR 1.52 [95% CI 1.30;1.78]) and respiratory rate (OR 1.42 [1.17;1.71]) were associated with a positive test. Finally, the decision to admit to hospital was associated with a positive test for SARS-CoV-2 (OR 2.78 [95% CI 1.32;5.43]) 

For all hospitalized patients (*n* = 41, related and unrelated to COVID-19) the most common reported symptoms were cough and exhaustion (26 patients, 63.4%), followed by measured/reported fever (23 patients, 56.1%) and headache (22 patients, 53.7%). Higher PDSR (OR 10.2 [95% CI 5.05;23.00]), seizure (OR 6.39 [95% CI 1.84;16.50]), nausea (OR 4.97 [95% CI 2.62;9.27]), confusion (OR 2.79 [95% CI 1.28;5.55]), dyspnea (OR 2.76 [95% CI 1.47;5.14]), and weakness (OR 2.73 [95% CI 1.44;5.49]) were most strongly associated with hospitalization. Regarding comorbidities, renal disease (OR 13.3 [95% CI 5.20; 29.40] and diabetes (OR 10.6 [4.83; 21.40]) were associated with hospitalization (Table A2).

Among all 572 SARS-CoV-2 positive tested presentations, 561 (98.1%) were discharged from the TTC (*n* = 538) and the ED (*n* = 23). All were immediately quarantined by the local health authorities. The most common diagnosis in patients discharged from the ED was presumed viral infection tested negative for SARS-CoV-2 (102 of 191 patients, 53.4%). Regarding hospitalization, COVID-19 was the leading cause (14 of 41 patients, 34.1%). Of note, in 11 out of 14 (positive) hospitalized patients, testing for SARS-CoV-2 was positive after the first swab. In 3 patients, testing was initially negative and repeated testing revealed a positive result during hospitalization (Table 2).

## 4. Discussion

The main results of our study indicate safety, feasibility, and the excellent clinical outcomes in a low-threshold Triage and Test Center for SARS-CoV-2. Safety was shown by a low re-presentation rate and low mortality during follow-up. Outcomes were excellent, as documented by low hospitalization and ED transfer rates. Feasibility was shown by the successful implementation of the process based on informal triage, the ability to provide a high throughput employing medical students as “swab-team” [9], and a low overruling rate. Analysis of clinical predictors showed that it is virtually impossible to include or exclude COVID-19 on clinical grounds only. Even though certain markers defining influenza-like illness, such as fever, myalgia, chills, and cough, were somewhat predictive of positive swab results in our setting, nonspecific symptoms, such as weakness and confusion [16] were similarly predictive. Although we previously demonstrated that nonspecific symptoms such as weakness are associated with unfavorable prognosis [17], these symptoms do not generally increase the probability of infection in (older) adults in the ED [18,19]. These symptoms appear to be unable to distinguish COVID-19 from other (infectious) illness. Therefore, clinical prediction based on symptoms and comorbidities alone cannot replace molecular diagnostic testing, and repeated testing may be indicated in patients with symptoms, but initially negative PCR test results. Low-threshold testing appears to be useful in order to identify cases that might otherwise have gone undetected. Chameleons, i.e., presentations that do not appear to be positive initially, but later turn out to be COVID-19 are not infrequent [20]. Conversely, COVID-19-mimics, such as the examples provided in Table 2 (apart from patients with COVID-19), accounted for almost two-thirds of hospitalized patients. Data from a large prospective cohort study showed that patients hospitalized for COVID-19 usually presented with fever, cough, and shortness of breath, as well as met the world health organization (WHO) case definitions for COVID-19 [21], but a control group was lacking. Similarly, in a large meta-analysis based on retrospective studies, cough was present only in 58% patients, which is similar to our study [22]. Dyspnea and fever, however, occurred less frequently in our setting, which is mostly due to inclusion of a healthier outpatient population. Another multicenter study developed a clinical score at hospital admission for predicting critical illness. In that study, dyspnea, hemoptysis, and unconsciousness were clinical predictors of critical illness [23]. While several clinical predictors for severe COVID-19 (dyspnea [24], old age [25], type II diabetes [26], obesity [27], possibly hypertension [28], history of cancer [29], cardiovascular disease [30], or increased ratios of CD4/CD8 [31], neutrophils/lymphocytes [32], and CRP/lymphocytes [33]) have been identified, no study has assessed clinical predictors for SARS-CoV-2 positivity. This knowledge is important for the upcoming phase, when the ratio of patients affected to patients tested will remain low in most test centers. The advantage of our approach, waiving formal triage, vital sign assessment, and physician examination in most patients is the reduction in resources used, while safety and feasibility are given. We speculate that, when effectively implemented, a low-threshold approach using a TTC may contribute to subduing the COVID-19 pandemic. This, however, will only be among a wide range of interventions, such as social distancing, contact tracing, and self-isolation of individuals tested positive for SARS-CoV-2.

## 5. Limitations

This study had several limitations. First, this is a single center study with limited external validity. Second, patients presented with symptoms were considered to be attributable to COVID-19. A self-referral bias appears likely. Third, as our patients were walk-in patients at the TTC, it must be assumed that this is a rather healthy population, which could cause a possible spectrum bias [34]. Verification bias appears to be minimal in our study, as almost all patients presenting to the TTC received RT-PCR testing. Fourth, patients filled out a questionnaire regarding symptoms and comorbidities by themselves. In 86 cases, for example, seizure was reported as a symptom. Therefore, response bias is likely. Fifth, we did not systematically assess hyposmia/anosmia or hypogeusia/ageusia as a predictor of COVID-19 positivity [35], as these predictors were not generally accepted on March 8th, when the study and the test process was designed. Sixth, we did not formally assess reasons for overruling. It can be speculated that patients who were deemed low risk (PDSR ≤ 2) but reported symptoms prompting further evaluation (e.g., dyspnea, chest pain) had their vital signs taken and were evaluated by medical doctor in charge. Seventh, we relied upon RT-PCR as the criterion standard for the diagnosis of COVID-19, which is an imperfect gold standard. The possibility of false-negative or false-positive RT-PCR results needs to be considered, especially as the pre-test probability or estimated risk of disease before testing was not formally assessed in our study [36], but was rather low. Moreover, the false-negative rate appears to be dependent on symptom duration [37] and the pre-analytical quality of the swabs. Our procedure to routinely combine nasopharyngeal with oropharyngeal swabs into one transport medium (as NOPS) is likely to increase the preanalytical quality of sampling and to reduce the number of false negatives. This is also supported by our two-target approach showing 97.5% to 99% concordance reflecting the very high SARS-CoV-2 loads in the transport medium of 10 million copies/mL (median 7.2 log_10_ copies/mL, IQR 5.8–8.4). Eighth, 13 COVID-19 positive patients were lost to follow-up. In addition, we could have missed other patients being tested positive at a later time point. However, as we are the only general public hospital in our Canton (State), and nearly all patients with possible COVID-19 symptoms presented or were referred to our hospital, other relevant missing data is unlikely, but possible. Family physicians, at that time, were not equipped with sufficient personal protective gear and were therefore reluctant to see COVID-19 patients. Further, local health authorities had a tight control on all cases who were tested positive. Finally, national health authorities did not endorse the low-threshold testing; instead, testing was recommended for patients with severe symptoms only. However, the overall positivity rates were comparable to a national average of roughly 14% in the period of testing [38].

## 6. Conclusions

Our study shows that installing a low-threshold walk-in TTC near the ED of a tertiary care hospital appears to be safe and is feasible. A clinical diagnosis of COVID-19 appears virtually impossible, given the only moderate associations of clinical predictors with SARS-CoV-2 positive PCR test results.

## Figures and Tables

**Table 1:** Baseline demographic and clinical characteristics of all included presentations.

	All (*n* = 4815)	Positive Swab (*n* = 572)	Negative Swab (*n* = 4243)	OR [95% CI]	*p*-Value
Age (years, median [min/max])	41.6 [13.4;93.3]	45.7 [16.5; 86.8]	41.2 [13.4;93.3]	1.31 [1.15;1.50]	<0.001
Male	2188 (45.4%)	289 (50.5%)	1899 (44.8%)	Ref.	Ref.
Female	2627 (54.6%)	283 (49.5%)	2344 (55.2%)	0.79 [0.67;0.94]	0.009
PDSR ≤2 PDSR >2	3563 (74%) 1252 (26%)	399 (69.8%) 173 (30.2%)	3164 (74.6%) 1079 (25.4%)	Ref. 1.18 [0.97;1.43]	Ref. 0.104
**Symptoms**					
Duration of symptoms (days, median [min/max]) ^1^	4 [1;119]	5 [1;55]	4 [1;119]	0.94 [0.89;1.00]	0.052
Measured/reported fever	2012 (41.8%)	323 (56.5%)	1689 (39.8%)	2.03 [1.70;2.42]	<0.001
Chills ^2^	1025 (23.8%)	165 (33.3%)	860 (22.6%)	1.77 [1.44;2.16]	<0.001
Myalgia	1859 (38.6%)	301 (52.6%)	1558 (36.7%)	1.94 [1.63;2.31]	<0.001
Lymphadenopathy	491 (10.2%)	33 (5.8%)	458 (10.8%)	0.54 [0.37;0.76]	<0.001
Headache	2693 (55.9%)	366 (64%)	2327 (54.8%)	1.61 [1.34;1.94]	<0.001
Seizure	86 (1.79%)	9 (1.57%)	77 (1.81%)	0.83 [0.38;1.57]	0.592
Confusion	509 (10.6%)	76 (13.3%)	433 (10.2%)	1.39 [1.06;1.80]	0.014
Nausea	668 (13.9%)	82 (14.3%)	586 (13.8%)	1.11 [0.86;1.43]	0.403
Conjunctivitis	469 (9.7%)	51 (8.9%)	418 (9.9%)	0.87 [0.64;1.17]	0.377
Exanthema	244 (5.1%)	20 (3.5%)	224 (5.3%)	0.66 [0.40;1.03]	0.081
Coryza	2575 (53.5%)	314 (54.9%)	2261 (53.3%)	1.10 [0.92;1.32]	0.279
Otalgia	749 (15.6%)	70 (12.2%)	679 (16%)	0.77 [0.59;1.00]	0.055
Sore throat	2694 (56%)	247 (43.2%)	2447 (57.7%)	0.59 [0.49;0.70]	<0.001
Dyspnea	1158 (24%)	101 (17.7%)	1057 (24.9%)	0.65 [0.52;0.82]	<0.001
Wheezing	675 (14%)	42 (7.3%)	633 (14.9%)	0.44 [0.32;0.61]	<0.001
Cough	3132 (65%)	415 (72.6%)	2717 (64%)	1.50 [1.24;1.83]	<0.001
Productive cough	1119 (23.2%)	132 (23.1%)	987 (23.3%)	0.97 [0.78;1.19]	0.743
Hemoptysis	53 (1.1%)	4 (0.7%)	49 (1.2%)	0.61 [0.18;1.51]	0.345
Chest pain	1335 (27.7%)	139 (24.3%)	1196 (28.2%)	0.83 [0.68;1.02]	0.075
Abdominal pain	664 (13.8%)	63 (11%)	601 (14.2%)	0.79 [0.59;1.03]	0.087
Diarrhea	842 (17.5%)	112 (19.6%)	730 (17.2%)	1.21 [0.97;1.51]	0.093
Dysuria	184 (3.8%)	22 (3.9%)	162 (3.8%)	0.99 [0.61;1.53]	0.981
Exhausted	2131 (44.3%)	271 (47.4%)	1860 (43.8%)	1.18 [0.98;1.40]	0.073
Weakness ^2^	1901 (44.1%)	255 (51.4%)	1646 (43.2%)	1.46 [1.21;1.76]	<0.001
**Comorbidities**					
Regular medication	1695 (35.2%)	190 (33.2%)	1505 (35.5%)	0.81 [0.66;0.98]	0.030
Neurologic disease	112 (2.3%)	7 (1.2%)	105 (2.5%)	0.45 [0.19;0.90]	0.040
Hypertension	648 (13.5%)	84 (14.7%)	564 (13.3%)	0.91 [0.70;1.19]	0.501
Diabetes	152 (3.2%)	31 (5.4%)	121 (2.9%)	1.64 [1.07;2.45]	0.019
Overweight	705 (14.6%)	82 (14.3%)	623 (14.7%)	0.90 [0.70;1.16]	0.428
Heart disease	232 (4.8%)	32 (5.6%)	200 (4.7%)	0.97 [0.65;1.43]	0.898
Pulmonary disease	221 (4.6%)	9 (1.6%)	212 (5%)	0.27 [0.13;0.50]	<0.001
Asthma	607 (12.6%)	51 (8.9%)	556 (13.1%)	0.66 [0.48;0.89]	0.007
Chronic obstructive pulmonary disease	67 (1.4%)	4 (0.7%)	63 (1.5%)	0.38 [0.11;0.93]	0.062
Renal disease	81 (1.7%)	15 (2.6%)	66 (1.6%)	1.54 [0.84;2.66]	0.138
Liver disease	86 (1.8%)	10 (1.8%)	76 (1.8%)	0.85 [0.41;1.58]	0.627
Hematological disease	39 (0.81%)	5 (0.9%)	34 (0.8%)	1.03 [0.35;2.42]	0.958
HIV infection	31 (0.6%)	5 (0.9%)	26 (0.6%)	1.27 [0.43;3.06]	0.631
Rheumatological disease	231 (4.8%)	26 (4.6%)	205 (4.8%)	0.82 [0.53;1.24]	0.373
Cancer	118 (2.5%)	16 (2.8%)	102 (2.4%)	0.97 [0.54;1.62]	0.910
**Vital signs**					
Systolic blood pressure (mmHg, median [min/max]) ^3^	141 [95;222]	139 [97;197]	142 [95;222]	1.38 [1.12;1.70]	0.002
Diastolic blood pressure (mmHg, median [min/max]) ^3^	82 [43;130]	81 [46;130]	82 [43;120]	1.21 [1.01;1.44]	0.036
Heart rate (beats per minute, median [min/max]) ^4^	83 [44;141]	85 [44;130]	83 [47;141]	1.24 [1.03;1.48]	0.019
Respiratory rate (breath per minute, median [min/max]) ^5^	17 [6;33]	17 [7;32]	16 [6;33]	1.42 [1.17;1.71]	<0.001
Oxygen saturation (median [min/max]) ^6^	98 [90;100]	97 [92;100]	98 [90;100]	1.06 [0.96;1.17]	0.275
Temperature (°C, median [min/max]) ^6^	36.8 [35;39.5]	36.9 [35.2;39.1]	36.7 [35;39.5]	1.52 [1.30;1.78]	<0.001

^1^ data available for 4402 presentations; ^2^ data available for 4307 presentations; ^3^ data available for 1788 presentations; ^4^ data available for 1797 presentations; ^5^ data available for 1782 presentations; ^6^ data available for 1798 presentations; OR = Odds Ratio; CI = Confidence Interval; Ref. = Reference; PDSR = Physician Disease Severity Rating.

**Table 2:** Diagnoses of Triage and Test Center (TTC) presentations.

**Diagnoses of COVID-19 in Presentations discharged from TTC (*n* = 4583)**	
Discharged with PDSR ≤2 (*n* = 3509)	392 positive testing for SARS-CoV-2
Discharged with PDSR >2 (*n* = 1074)	146 positive testing for SARS-CoV-2
**Diagnoses of hospitalized patients with PDSR ≤2 (*n* = 9)**	
Number of patients	Diagnosis
2	COVID-19 ^1^
1	Anorectal abscess
1	Behavioral disorder
1	Pneumonia
1	Hepatitis
1	Atraumatic Pneumomediastinum
1	Sinusitis
1	Screening before planned hospital admission for vocal cord biopsy
**Diagnoses of patients with PDSR ≤2 discharged from ED (*n* = 45)**	
Number of patients	Diagnosis
6	COVID-19
24	Presumed viral infection tested negative for SARS-CoV-2
5	Chest pain of unclear origin
2	Asthma
2	Dyspnea of unclear origin
1	Hypertensive urgency
1	Diarrhea
1	Urinary tract infection
1	Abdominal pain of unclear origin
1	Cocaine intoxication
1	Behavioral disorder
**Diagnoses of hospitalized patients with PDSR >2 (*n* = 32)**	
Number of patients	Diagnosis
12	COVID-19 ^2^
6	Pneumonia
2	Coronary heart disease
2	Presumed viral infection tested negative for SARS-CoV-2
1	Decompensated heart failure
1	Perimyocarditis
1	Pulmonary embolism
1	Asthma
1	Angina
1	Appendicitis
1	Urinary tract infection
1	Pyelonephritis
1	Wound infection
1	Behavioral disorder
**Diagnoses of patients with PDSR >2 discharged from ED (*n* = 146)**	
Number of patients	Diagnosis
17	COVID-19
78	Presumed viral infection tested negative for SARS-CoV-2
14	Chest pain of unclear origin
12	Asthma
5	Angina
3	Hypertensive urgency
3	Urinary tract infection
2	Abdominal pain of unclear origin
2	Pneumonia
2	Dyspnea of unclear origin
1	Pulmonary embolism
1	Chronic cough of unclear origin
1	Exacerbation of chronic obstructive pulmonary disease
1	Laryngitis
1	Dyspepsia
1	Pyelonephritis
1	Traumatic subarachnoid hemorrhage
1	Behavioral disorder

^1^ in 1 patient testing for SARS-CoV-2 was negative on admission and turned positive during hospitalization; ^2^ in 2 patients testing for SARS-CoV-2 was negative on admission and turned positive during hospitalization; COVID-19 = coronavirus disease 19; PDSR = Physician Disease Severity Rating; SARS-CoV-2 = severe acute respiratory syndrome coronavirus 2; ED = Emergency Department.

## Appendix A

**Table A1:** Overruling of patient presentations.

PDSR	Total Presentations	Vitals Measured	Vitals not Neasured
PDSR 1	2041	223 ^1^	1818 ^2^
PDSR 2	1522	346 ^3^	1176 ^4^
**PDSR ≤2**	**3563**	**569 (16%)**	**2994 (84%)**
PDSR 3	1055	1037 ^5^	18 ^6^
PDSR 4	155	151 ^7^	4 ^8^
PDSR 5	36	35 ^9^	1 ^10^
PDSR 6	6	6 ^11^	0
**PDSR >2**	**1252**	**1229 (98.2%)**	**23 (1.8%)**

^1^ of which 9 were transferred to ED for further work-up, 9 outpatient treatment; ^2^ of which 5 were transferred to ED for further work-up, 3 outpatient treatment, 2 hospitalized; ^3^ of which 38 were transferred to ED for further work-up, 32 outpatient treatment, 6 hospitalized; ^4^ of which 2 were transferred to ED for further work-up, 1 outpatient treatment, 1 hospitalized; ^5^ of which 121 were transferred to ED for further work-up, 105 outpatient treatment, 16 hospitalized; ^6^ of which 3 were transferred to ED for further work-up, 1 outpatient treatment, 2 hospitalized; ^7^ of which 39 were transferred to ED for further work-up, 31 outpatient treatment, 8 hospitalized; ^8^ of which 3 were transferred to ED for further work-up, 1 outpatient treatment, 2 hospitalized; ^9^ of which 9 were transferred to ED for further work-up, 6 outpatient treatment, 3 hospitalized; ^10^ of which 1 were transferred to ED for further work-up, 1 outpatient treatment; ^11^ of which 2 were transferred to ED for further work-up, 1 outpatient treatment, 1 hospitalized; PDSR = Physician Disease Severity Rating; ED = Emergency Department.

**Table A2:** Characteristics stratified by disposition status for all presentations (related and unrelated to COVID-19).

	All (*n* = 4815)	Discharged (*n* = 4774)	Hospitalized (*n* = 41)	OR [95% CI]	*p*-Value
Age (years, median [min;max])	41.6 [13.4;93.3]	41.4 [13.4;93.3]	62.1 [21.2;90.7]	1.08 [1.06;1.10]	<0.001
Male	2188 (45.4%)	2162 (45.3%)	26 (63.4%)	Ref.	Ref.
Female	2627 (54.6%)	2612 (54.7%)	15 (36.6%)	0.48 [0.25;0.90]	0.022
PDSR≤2 PDSR>2	3563 (74%) 1252 (26%)	3554 (74.4%) 1220 (25.6%)	9 (22.0%) 32 (78%)	Ref. 10.2; [5.05;23.00]	Ref. <0.001
**Symptoms**					
Duration of symptoms (days, median [min;max]) ^1^	4 [1;119]	4 [1;119]	6.5 [1;27]	1.01 [0.98;1.04]	0.671
Measured/reported fever	2012 (41.8%)	1989 (41.7%)	23 (56.1%)	1.79 [0.96;3.37]	0.067
Chills ^2^	1025 (23.8%)	1008 (23.6%)	17 (41.5%)	2.30 [1.20;4.28]	0.012
Myalgia	1859 (38.6%)	1840 (38.5%)	19 (46.3%)	1.38 [0.73;2.56]	0.313
Lymphadenopathy	491 (10.2%)	484 (10.1%)	7 (17.1%)	1.86 [0.74;3.98]	0.170
Headache	2693 (55.9%)	2671 (55.9%)	22 (53.7%)	0.91 [0.49;1.71]	0.768
Seizure	86 (1.79%)	52 (1.7%)	4 (9.8%)	6.39 [1.84;16.5]	0.007
Confusion	509 (10.6%)	499 (10.5%)	10 (24.4%)	2.79 [1.28;5.55]	0.011
Nausea	668 (13.9%)	650 (13.6%)	18 (43.9%)	4.97 [2.62;9.27]	<0.001
Conjunctivitis	469 (9.7%)	465 (9.7%)	4 (9.8%)	1.04 [0.30;2.61]	0.946
Exanthema	244 (5.1%)	239 (5%)	5 (12.2%)	2.71 [0.91;6.38]	0.070
Coryza	2575 (53.5%)	2557 (53.6%)	18 (43.9%)	0.68 [0.36;1.26]	0.223
Otalgia	749 (15.6%)	745 (15.6%)	4 (9.8%)	0.61 [0.18;1.52]	0.313
Sore throat	2694 (56%)	2679 (56.1%)	15 (36.6%)	0.45 [0.23;0.85]	0.013
Dyspnea	1158 (24%)	1139 (23.9%)	19 (46.3%)	2.76 [1.47;5.14]	0.002
Wheezing	675 (14%)	664 (13.9%)	11 (26.8%)	2.29 [1.09;4.48]	0.031
Cough	3132 (65%)	3106 (65.1%)	26 (63.4%)	0.93 [0.49;1.80]	0.817
Productive cough	1119 (23.2%)	1104 (23.1%)	15 (36.6%)	1.93 [0.99;3.62]	0.054
Hemoptysis	53 (1.1%)	52 (1.1%)	1 (2.4%)	2.58 [0.11;12.1]	0.440
Chest pain	1335 (27.7%)	1324 (27.7%)	11 (26.8%)	0.96 [0.46;1.88]	0.919
Abdominal pain	664 (13.8%)	653 (13.7%)	11 (26.8%)	2.33 [1.11;4.56]	0.027
Diarrhea	842 (17.5%)	833 (17.4%)	9 (22%)	1.35 [0.60;2.73]	0.448
Dysuria	184 (3.8%)	180 (3.8%)	4 (9.8%)	2.85 [0.83;7.24]	0.089
Exhausted	2131 (44.3%)	2105 (44.1%)	26 (63.4%)	2.19 [1.17;4.26]	0.014
Weakness ^2^	1901 (44.1%)	1873 (43.9%)	28 (68.3%)	2.73 [1.44;5.49]	0.002
**Comorbidities**					
Regular medication	1695 (35.2%)	1673 (35%)	22 (53.7%)	2.14 [1.15;4.02]	0.016
Neurologic disease	112 (2.3%)	109 (2.3%)	3 (7.3%)	3.54 [0.82;10.0]	0.084
Hypertension	648 (13.5%)	638 (13.4%)	10 (24.4%)	2.11 [0.97;4.20]	0.058
Diabetes	152 (3.2%)	142 (3%)	10 (24.4%)	10.6 [4.83;21.4]	<0.001
Overweight	705 (14.6%)	693 (14.5%)	12 (29.3%)	2.46 [1.19;4.73]	0.016
Heart disease	232 (4.8%)	228 (4.8%)	4 (9.8%)	2.23 [0.65;5.64]	0.178
Pulmonary disease	221 (4.6%)	218 (4.6%)	3 (7.3%)	1.73 [0.40;4.84]	0.407
Asthma	607 (12.6%)	604 (12.7%)	3 (7.3%)	0.57 [0.13;1.59]	0.317
Chronic obstructive pulmonary disease	67 (1.4%)	67 (1.4%)	0 (0%)		
Renal disease	81 (1.7%)	74 (1.6%)	7 (17.1%)	13.3 [5.20;29.4]	<0.001
Liver disease	86 (1.8%)	84 (1.8%)	2 (4.9%)	3.07 [0.46;10.2]	0.020
Hematological disease	39 (0.81%)	37 (0.8%)	2 (4.9%)	7.01 [1.03;24.2]	0.047
HIV infection	31 (0.6%)	31 (0.6%)	0 (0%)		
Rheumatological disease	231 (4.8%)	229 (4.8%)	2 (4.9%)	1.09 [0.16;3.59]	0.907
Cancer	118 (2.5%)	113 (2.4%)	5 (12.2%)	5.88 [1.96;14.0]	0.003
**Vital signs**					
Systolic blood pressure (mmHg, median [min;max]) ^3^	141 [95;222]	141 [95;222]	137 [102;165]	1.01 [0.99;1.03]	0.151
Diastolic blood pressure (mmHg, median [min;max])^3^	82 [43;130]	82 [43;130]	76.5 [47;106]	1.04 [1.01;1.07]	0.008
Heart rate (beats per minute, median [min;max]) ^4^	83 [44;141]	83 [44;141]	93 [65;136]	1.05 [1.02;1.07]	<0.001
Respiratory rate (breath per minute, median [min;max]) ^5^	17 [6;33]	17 [6;33]	20 [12;26]	1.23 [1.13;1.34]	<0.001
Oxygen saturation (median [min;max]) ^6^	98 [90;100]	98 [90;100]	96 [92;99]	1.89 [1.58;2.26]	<0.001
Temperature (°C, median [min;max]) ^6^	36.8 [35;39.5]	36.7 [35;39.5]	37.2 [36;39.5]	3.73 [2.44;5.71]	<0.001

^1^ data available for 4402 presentations, ^2^ data available for 4307 presentations, ^3^ data available for 1788 presentations, ^4^ data available for 1797 presentations, ^5^ data available for 1782 presentations, ^6^ data available for 1798 presentations; COVID-19 = coronavirus disease 19; OR = Odds Ratio; CI = Confidence Interval; Ref. = Reference; PDSR = Physician Disease Severity Rating.
